# X-ray Free Electron Laser Determination of Crystal Structures of Dark and Light States of a Reversibly Photoswitching Fluorescent Protein at Room Temperature

**DOI:** 10.3390/ijms18091918

**Published:** 2017-09-07

**Authors:** Christopher D. M. Hutchison, Violeta Cordon-Preciado, Rhodri M. L. Morgan, Takanori Nakane, Josie Ferreira, Gabriel Dorlhiac, Alvaro Sanchez-Gonzalez, Allan S. Johnson, Ann Fitzpatrick, Clyde Fare, Jon P. Marangos, Chun Hong Yoon, Mark S. Hunter, Daniel P. DePonte, Sébastien Boutet, Shigeki Owada, Rie Tanaka, Kensuke Tono, So Iwata, Jasper J. van Thor

**Affiliations:** 1 Molecular Biophysics, Imperial College London, South Kensington Campus, London SW7 2AZ, UK; christopher.hutchison05@imperial.ac.uk (C.D.M.H.); violeta.cordon-preciado@imperial.ac.uk (V.C.-P.); j.ferreira14@imperial.ac.uk (J.F.); gabriel.dorlhiac13@imperial.ac.uk (G.D.); c.fare12@imperial.ac.uk (C.F.); 2 Protein Crystallography Facility, Centre for Structural Biology, Flowers Building, Department of Life Sciences, Imperial College London, London SW7 2AZ, UK; rhodri.morgan@imperial.ac.uk; 3 Department of Biological Sciences, Graduate School of Science, The University of Tokyo, 2-11-16 Yayoi, Bunkyo-ku, Tokyo 113-0032, Japan; takanori.nakane@bs.s.u-tokyo.ac.jp; 4 Quantum Optics and Laser Science Group, Blackett Laboratory, Imperial College, London SW7 2AZ, UK; a.sanchez-gonzalez13@imperial.ac.uk (A.S.-G.); allan.s.johnson@gmail.com (A.S.J.); j.marangos@imperial.ac.uk (J.P.M.); 5 Diamond Light Source Ltd., Diamond House, Harwell Science & Innovation Campus, Didcot OX11 0DE, UK; ann.fitzpatrick@diamond.ac.uk; 6 LCLS, SLAC National Accelerator Laboratory, 2575 Sand Hill Rd., Menlo Park, CA 94025, USA; yoon82@stanford.edu (C.H.Y.); mhunter2@slac.stanford.edu (M.S.H.); deponte@slac.stanford.edu (D.P.D.); sboutet@slac.stanford.edu (S.B.); 7 RIKEN SPring-8 Center, 1-1-1 Kouto, Sayo-cho, Hyogo 679-5148, Japan; osigeki@spring8.or.jp (S.O.); rie.tanaka.hw@riken.jp (R.T.); tono@spring8.or.jp (K.T.); s.iwata@spring8.or.jp (S.I.); 8 Japan Synchrotron Radiation Research Institute, 1-1-1 Kouto, Sayo-cho, Hyogo 679-5198, Japan; 9 Department of Cell Biology, Graduate School of Medicine, Kyoto University, Yoshidakonoe-cho, Sakyo-ku, Kyoto 606-8501, Japan

**Keywords:** XFEL, SFX, rsFP, Skylan-NS, SACLA, LCLS

## Abstract

The photochromic fluorescent protein Skylan-NS (Nonlinear Structured illumination variant mEos3.1H62L) is a reversibly photoswitchable fluorescent protein which has an unilluminated/ground state with an anionic and cis chromophore conformation and high fluorescence quantum yield. Photo-conversion with illumination at 515 nm generates a meta-stable intermediate with neutral trans-chromophore structure that has a 4 h lifetime. We present X-ray crystal structures of the cis (on) state at 1.9 Angstrom resolution and the trans (off) state at a limiting resolution of 1.55 Angstrom from serial femtosecond crystallography experiments conducted at SPring-8 Angstrom Compact Free Electron Laser (SACLA) at 7.0 keV and 10.5 keV, and at Linac Coherent Light Source (LCLS) at 9.5 keV. We present a comparison of the data reduction and structure determination statistics for the two facilities which differ in flux, beam characteristics and detector technologies. Furthermore, a comparison of droplet on demand, grease injection and Gas Dynamic Virtual Nozzle (GDVN) injection shows no significant differences in limiting resolution. The photoconversion of the on- to the off-state includes both internal and surface exposed protein structural changes, occurring in regions that lack crystal contacts in the orthorhombic crystal form.

## 1. Introduction

Photochromic fluorescent proteins that are reversibly photoswitchable have been reported for constructs and mutants derived from different origins [1,2,3,4,5,6,7,8,9]. When focusing on the class of reversibly photoswitchable fluorescent proteins (rsFP’s) all involve cis-trans and trans-cis photoisomerisation reactions. While characteristics such as protein structural response, photoswitching quantum yields, fluorescence quantum yields of cis and trans chromophore conformations all differ among the members of rsFP’s the definitive switching reaction remains the same. A prototype rsFP with characteristics very similar to those reported here and representative of several other reports is the ‘Dronpa’ construct from the coral *Pectiniidae* [4]. With few exceptions [10], this class of photochromic rsFPs have a dark/resting state with generally a high fluorescence quantum yield and a cis chromophore conformation which is anionic at neutral or alkaline pH [11]. Following green illumination this ‘on’ state converts to a meta-stable off-state which has a trans chromophore conformation and is neutral. The photochemical quantum yield of cis-trans (on→off) photoisomerisation differs for reported mutants and constructs, but are generally at the few percent level for the fastest switching cases [12,13,14]. The off-state typically absorbs blue light, at 390 nm in the case of Dronpa [4]. Blue illumination triggers trans-cis (off→on) photoisomerisation with generally a highly enhanced photochemical quantum yield, in the case of Dronpa reported at ≈30% [15]. In the dark at room temperature the off-state converts thermally to the resting ‘on’ state with typical time constant of minutes to hours depending on the details of the construct [12].

The measurement of the ‘on’ [16,17] and ‘off’ [18] structures of Dronpa revealed a reorientation of the Arg-66 and His-193 side chains, in addition to the chromophore movement. Ultrafast spectroscopy of the fast switching Dronpa mutant M159T [15] showed the formation of the primary photoproduct occurs with time constants of 1.9, 185, and 1089 ps for the on→off reaction and 0.6 and 14 ps for off→on. The mGeos [12] series of rsFP’s display similar switching behavior to Dronpa. The series was developed from mEos2 [19], with mutations that remove the undesirable irreversible green-to-red photo conversation, increase fluorescence brightness and provide high thermal stability of the off-state [12]. Several constructs of the mGeos series have been shown to have a faster off→on switching rate than that of Dronpa. Skylan-NS represents a further improvement, with reduced per cycle bleaching and higher photoswitching quantum yield in the on→off than Dronpa [13].

Hard X-ray free electron lasers (XFELs) have proven to be a powerful tool to collect crystallographic data from protein crystals using the Serial Femtosecond Crystallography (SFX) method [20,21]. In contrast to synchrotron methods, SFX performed at XFELs allows protein structures to be collected at room temperature while remaining almost radiation damage free [22,23]. A concern in the XFEL community is the comparability of SFX data collected at different facilities due to the large number of variables that can impact crystal quality and reproducibility. We present high resolution room temperature SFX structures of Skylan-NS in both ‘on’ and meta-stable ‘off’ state collected using two different XFELs, the SPring-8 Angstrom Compact free electron Laser (SACLA), Japan and the Linear Coherent Light Source (LCLS), USA. We also compare different SFX crystal injection methods and demonstrate the reliability of assigned light induced differences against those associated with different injection methods and XFELs. Three separate methods were used to deliver room temperature micro-crystals into the XFEL interaction region: a grease injector [24] and Droplet injector [25] at SACLA (Experimental Hutch 4 (EH4) 2016A8041 and EH2 2016A8032), and a Gas Dynamic Virtual Nozzle (GDVN) [26] at LCLS (Coherent X-ray Imaging instrument (CXI) run 15; PCS).

Preliminary crystallographic tests were performed at the Diamond Light Source (Rutherford Appleton Labs) synchrotron on crystals of Skylan-NS. These demonstrated that high resolution (<1.6 Å) could be collected and that complete photoswitching in crystal form was possible without significant loss of resolution or diffraction quality as judged from statistics. The light induced differences between the on and off states therefore present a method to create light induced isomorphous structure factor differences that can be evaluated with regard to crystallographic accuracy arising from different SFX conditions and methods.

## 2. Results

### 2.1. SFX Data (SACLA and LCLS)

We collected datasets for the dark on-state using two different methods at the SACLA/EH4 station using the DAPHNIS chamber [27]. Firstly, by using the grease injection method, we selected relatively large crystals of 50–100 μm in size. The SACLA pipeline was used for hit selection and processing. Diffraction was collected up to 1.9 Å resolution with diffraction still present in the corners of the detector. The point group was determined to be 222 and a molecular replacement in space group P212121 using Phaser [28] (Z-score 7.86) or Molrep (9.97 sigma) was done using a search model created from Dronpa [PDB code:2GX2] including additional mutations. For comparison, we obtained complementary data using the droplet-on-demand method for the dark on-state, using smaller crystals than those used for the grease method, which were delivered in the crystallization mother liquor. The hit-rate could be carefully controlled with the crystal density, and levels of 40% could be sustained with crystal densities of 4.8 × 10^7^–9 × 10^7^ crystal/mL. Table 1 shows the CC* [29] and R-split [30] values that reveal data from the droplet method was lower quality than that obtained from the grease injection method. Where CC* $=\;\sqrt{2{\mathrm{CC}}_{1/2}/1+{\mathrm{CC}}_{1/2}}$ and CC_1/2_ is the Pearson correlation coefficient between random half-datasets [29]. Scaling and merging the grease injection and droplet injection intensities together degraded the CC* and R-split values, although unit cell dimensions refined to the same values. Further investigations following scaling the individual datasets indicated genuine differences between the structures (see below). Therefore, both on-state datasets were processed separately.

**Figure 1:**
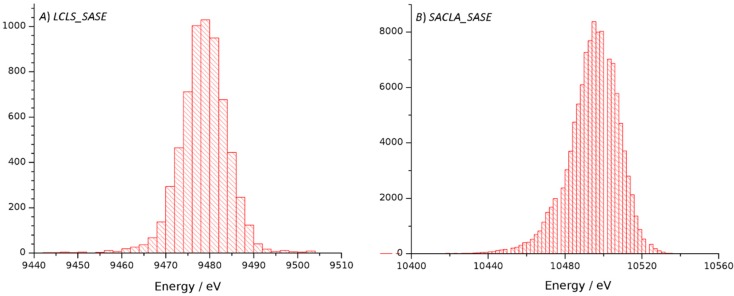
Histograms for the distribution of mean energy measured during data collection at LCLS/CXI (**A**) and SACLA/EH2 (**B**). Bin widths are 2 eV for both histograms.

In order to investigate the origin of the weaker diffraction in the case of the droplet data we evaluated and compared measurements made with the GVDN method [26] using higher photon energy and detector distances which support the observation of higher resolution diffraction down to 1.5 Angstroms. Both the droplet method as well as the GVDN method showed diffraction in the corners of the detectors to this resolution, indicating that the pressure wave that launches the droplet on demand does not significantly affect the resolution supported by crystal ordering (Table 1). As the GVDN data was collected at LCLS/CXI using a different detector and source as compared to the droplet data collected at SACLA/EH4 and SACLA/EH2, the processing statistics in terms of data strength, scaling and merging statistics as well as refinement statistics provide a comparison between the two facilities. With regard to the source, the main differences were different photon energies but also fluctuations of mean energies. At LCLS, a mean energy of 9478.7 eV was reported with a standard deviation of 6.6 eV, whereas SACLA provided a mean energy of 10,494.5 eV and a standard deviation of 13.5 eV (Figure 1). While the fluctuations are greater for the SACLA source, they are both within the ΔE/E which for the self-amplified spontaneous emission (SASE) mode are given as ≈1 × 10^−3^ for SACLA [31] and ≈ 4 × 10^−4^ LCLS [32].

The second significant difference is the detector used for data-collection. At SACLA, the multi-port charge-coupled device (MPCCD) detector [33], with simple and reliable geometry definitions, provided high signal to noise measurements. Prior tests using a low-resolution mask to attenuate intense reflections did not show improve data quality and it was not used for the presented data. Furthermore, profile fitting was also not used, while overloads were occasionally observed. At LCLS, we used the Cornell-SLAC Pixel Array Detector (CSPAD) [34,35,36,37,38], a silicon based sensor which has the ability to apply electronic gain masks to attenuate the low order reflections [37]. As reported previously [39,40] the dynamic range of hybrid gain mode (103-104) was found to be comparable to that of the charge coupled-device (CCD) detector used at SACLA [33], resulting in overall similar data reduction statistics (Table 1).

**Figure 2:**
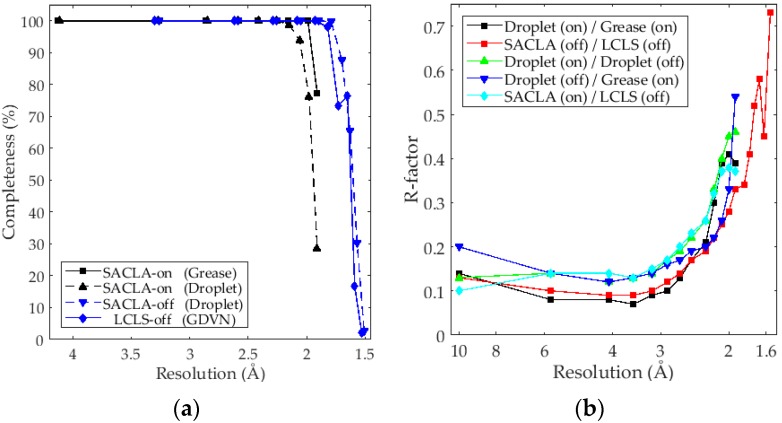
(**a**) Completeness of merged datasets shown in Table 1; (**b**) R-factors resulting from scaling by normal probability analysis for datasets in Table 1 of on- and off- state experiments at SACLA and LCLS.

Monte-Carlo integration using the “Partialator” binary [30] with unity model proved to give the best quality reduced and merged intensities. Attempts at using post-refinement of partiality [41,42,43] did improve R-split and CC * but at the expense of too many rejected frames. Thus, data was merged without post refinement. The higher resolution datasets, both collected for the off-state after saturating green illumination and were readily phased by molecular replacement with 10.02 and 9.27 sigma solutions. No resolution cutoff was used for any dataset and high-resolution reflections collected in the corners were kept despite low completeness in these bins. This was done to demonstrate that crystals, flux and detectors could support the higher resolution, rather than choosing a lower resolution cutoff based on completeness. Figure 2a shows the completeness plotted as a function of resolution. It can be seen that all four datasets have full completeness to 2.2 Å. The two off-state datasets go to higher resolution, but mainly due to the higher photon energy and short detector distances used during these beam times.

After conversion of the intensities to structure factors, the two on-state and two off-state datasets were scaled together using normal probability analysis applying a single scale factor and isotropic B-factors [44]. Weights by standard deviations were not used for scaling as this caused incorrect scaling of data with differences on low resolution terms. Figure 2b shows the resulting R-factors.

The comparison between the two on-state structures collected with the grease injection and droplet methods under the same conditions (Figure 2b, Black) showed the lowest R-factor over the intermediate resolution range up to ≈3 Å resolution. An increase of the R-factor for the increased resolution (<3 Å) is likely indicative of genuine structural differences between the grease and droplet methods, in agreement with the observed degradation of R-split and CC* when merging intensities together (Figure 2b, Black). The dehydrating effect on the crystals due to the mother liquor being replaced with a mineral oil–based grease matrix in the grease injection method could explain this difference.

A remarkable agreement was found between the LCLS and SACLA datasets of the off-state for resolution below 2 Å, while the R-factor is also reasonable for the higher resolution terms (Figure 2b, Red). The R-factor for the on- and off-state experiments using the droplet methods (Figure 2b, green) (but at different photon energy) is considerably higher than the two off-state measurements conducted at different facilities, including the lower resolution reflections. This is an indication that the structural differences caused by photoconversion, considering the 100% level of conversion, contribute significantly to the structure factor differences. This was also verified by computing the R-factor for F_calc_ (on-state) and F_calc_ (off-state) from coordinates after complete refinement, which were at similar levels. Table 1 shows in addition the full refinement statistics of all four data sets.

**Figure 3:**
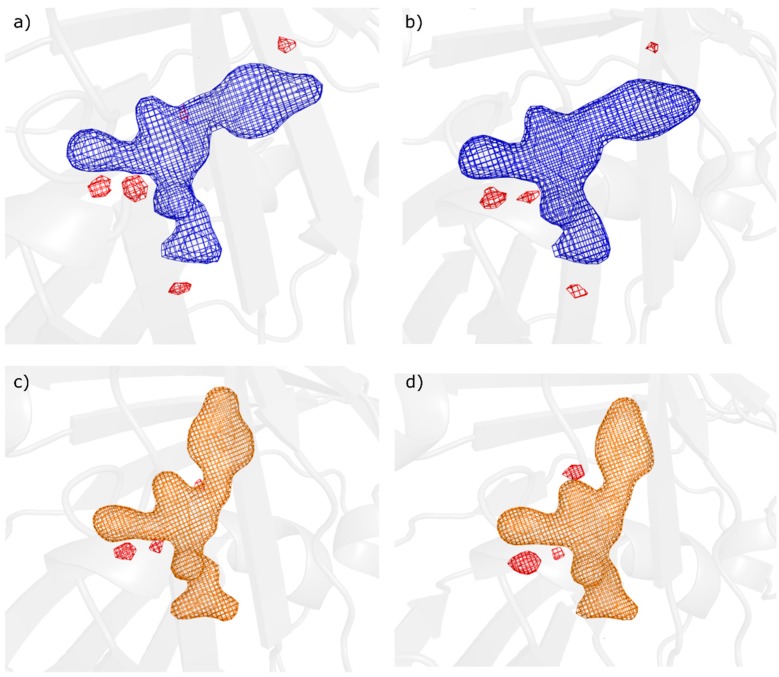
Chromophore omit maps, F_obs_-F_calc_ after full refinement plotted at 5 sigma level (+5σ Blue/−5σ Red for on-state; +5σ Orange/−5σ Red for off-state) for (**a**) On-state with grease injection at SACLA/EH4, (**b**) On-state with droplet injection at SACLA/EH4, (**c**) Off-state with droplet injection at SACLA/EH2 and (**d**) Off-state with GDVN injection at LCLS/CXI.

Next, we computed chromophore F_obs_-F_calc_ omit maps, which are presented in Figure 3. These indicated the complete photoisomerisation of the chromophore in the cis on-state and the trans off-state. The residual negative features present in all on- and off-state omit maps, as well as in the conventional F_obs_-F_calc_ maps after full refinement, likely indicate some residual positional errors [45,46] originating from the chromophore parameterization used in refinement. Unrestrained refinement however caused unrealistic chromophore distortion but did reduce the remaining features in the F_obs_-F_calc_ maps.

### 2.2. Structural Changes Resulting From Cis-Trans Photoisomerisation

Optical spectroscopy measurements of crystal slurry at pH 8.0 and room temperature showed that green illumination caused cis-trans photoisomerisation to proceed to completion, with only a very small fraction of the absorption of the cis state remaining. The time constant associated with thermal recovery was found to be 4–5 h.

Following complete refinement of all four datasets, both on-state structures showed no statistically significant density in either the 2F_obs_-F_calc_ or the F_obs_-F_calc_ maps that could be assigned to a trans chromophore structure. The same was observed for chromophore omit maps, that also, at lower sigma levels, did not show such density. This is in agreement with the dark incubation and dark sample injection which corresponds to a 100% on-state concentration. Similarly, both off-state structures were seen to have trans chromophore conformations with full occupancy, giving no indication for the presence of on-state with cis conformation in conventional and omit difference maps (Figure 3).

The refinement statistics of both on-state and off-state structures furthermore were in agreement with the data merging and scaling statistics (Table 1), which support the conclusion that complete photoconversion after intense green illumination (see methods) did not degrade the diffraction quality of the crystals by any criterion. Considering that optical measurements also showed negligible bleaching with full conversion, the off-state crystal structures are found to represent the pure photoisomerisation product and not a mixture of photo products. When the cis and trans data sets where combined the final RMS difference between the two refined models was 0.112 Å.

**Figure 4:**
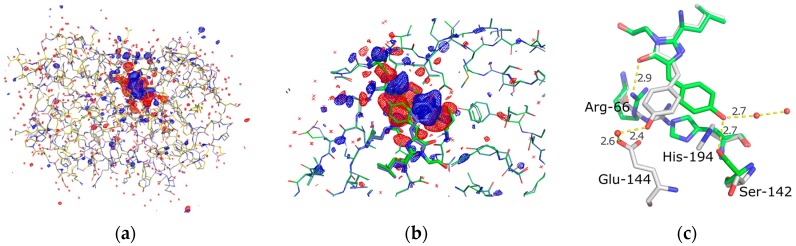
(**a**) F_obs_(on)-F_obs_(off) difference map (+3 sigma, blue/−3 sigma, red) shown for the entire protein; (**b**) A zoom of the chromophore region showing refined coordinates for the on- and off- states together with the light induced difference map; (**c**) A highlight of the structural differences in the on- (green) and off- (grey) states of the chromophore, key side chains and hydrogen bonds that display light induced differences are labeled.

Following scaling using the normal probability analysis procedure, the F_obs_(on)-F_obs_(off) difference map shows strong difference density features that are fully consistent with the changes of the refined coordinates following photoisomerisation (Figure 4). The resolution of the map is limited to that of the on-state at 1.9 A (Table 1). The strongest signals are at ≈10 sigma level and located on the chromophore phenolic ring and the histidine-194. At 3 sigma level all difference density signals are confined to the protein and do not extend significantly beyond (Figure 4a).

Photoisomerisation is clearly seen in a comparison of the “on” and “off” refined structures (Figure 4c) where the dihedral angle of the chain connecting the phenol ring to the rest of the chromophore (N–C=C–C) changes from 1° (cis) → 171.1° (trans) in the on- vs the off-state and the pheonolic oxygen is displaced by 6.2 Å. In this off-state the chromophore phenol ring has been repositioned to include an indirect hydrogen bonding via an ordered water to the carboxylate of glutamate-144. This arrangement is similar to the wild type green fluorescent protein from *Aequorea*, where a similar hydrogen bonding network exists that stabilizes the protonation of the phenolic oxygen of the chromophore [47]. In the off-state of Skylan-NS, the hydrogen bonding distances are 2.4 Å and 2.6 Å between the phenolic oxygen and water, and water and carboxylate respectively.

In addition to the chromophore movement several side chains inside the chromophore environment show large displacements. The side chains of the Ser-142 and His-194 undergo rotations. The former rotates about N-Cα-Cβ-OH from 66° → −164.6° and the later about N-C_α_-C_β_-C_γ_ from −70.6° → 113.4°. The Arg-66 side chain also displays a translation/extension motion where the direct hydrogen bonding between the guanidinium group and the oxygen of the chromophores’ imidazolic base can been disrupted and Arg-66 has been pulled “below” the chromophore by it binding to Glu-144 and the newly displaced His-194, resulting in a total movement of 2.6 Å towards His-194.

## 3. Discussion

The structures of Skylan-NS are very similar to that of Dronpa and other rsFP’s in this class, exhibiting rearrangement of residues in chromophore environment in addition to the cis-trans movement chromophore environment. One difference of note is that in contrast to the relatively small motion of Ser-142 in the case of photoconversion of Dronpa [18], in Skylan-NS the equivalent Ser-142 undergoes a more significant change of coordinates. In particular, the side chain oxygen moves by 4.1 Å after disruption of the hydrogen bonding to the chromophore phenolic oxygen in the on-state following photoconversion (Figure 4c). It is also noted that these and other changes that affect the surface exposed residues of the protein are not involved in crystal contacts. This could be significant considering the excellent diffraction after photoconversion, in contrast to Dronpa. Indeed, the solvent content of the orthorhombic crystal form of Skylan-NS was found to be ≈46% with considerable volumes between symmetry copies. The Matthews coefficient was found to be 2.26 Å^3^/Dalton.

Software, theory and methods developed for SFX are highly mature and optimized, and routine determination of very high quality structure factor amplitudes is also aided by instrumentation and source stability developments. At XFELs, however, challenges include the partiality arising from stationary diffraction and strong spectral structure which selects portions of the Ewald sphere differently for each pulse. The latter may be ameliorated to some extent by using self-seeded mode rather than SASE mode; however improvements in the resulting merging and scaling statistics have not yet been demonstrated [48]. Additionally, XFELs have jitter in intensity, mean photon energy, and pointing. Furthermore, diffractive beam delivery optics have been shown to add significant structure to the beam which, together with beam pointing jitter, are understood to add additional uncertainty to the structure factor amplitude determination [49]. Our present study is also in agreement with the view that Monte-Carlo approaches are currently favorable for many scenarios, particularly with relatively limited numbers of frames available. Post refinement of partiality has been reported however [50], and is in principle available in the CrystFEL package.

Here we show that data collected at two different XFEL facilities where the quality and accuracy are comparable. These facilities have different detector technologies, different source characteristics, and different sample injection methods (one of which involves the exposure of the crystals to a high-pressure pulse). The light induced differences between the on and off state of Skylan-NS were shown to be statistically larger than systematic experimental differences between datasets. This is a testament to the quality and maturity of XFEL source and beamline instrumentation, as well as to the maturation of software and crystallography methods, specifically for the Serial Femtosecond Crystallography technique [30,33,51,52].

## 4. Materials and Methods

### 4.1. Purification and Crystallization

A pRSETa plasmid with the Skylan-NS insert was obtained from Prof. Pingyong Xu’s laboratory. *E. coli* BL21(DE3) cells (New England Biolabs, Ipswich, MA, USA) were transformed with the recombinant plasmid and grown on non-inducing LB Ampicillin plates. A positive clone was selected, grown in LB Ampicillin media overnight and a 15% glycerol stock was kept corresponding to strain JVT41. For large-scale protein expression, a 70 L bioreactor (70 L ADI 1075 Pilot System with an ADI 1010 Bio Controller, Applikon Dependable Instruments, Delft, The Netherlands), with a 50 L working volume, was used. A 500 mL starter culture was grown overnight at 37 °C in LB supplemented with 1.5 g/L glycerol and 100 μg/mL carbenicillin. For the 50 L bioreactor culture, terrific broth media (EZmix^TM^, Sigma, St. Louis, MO, USA) supplemented with 8 g/L glycerol was used. The 50 L culture was inoculated with the starter culture, a corresponding dose of 100 μg/mL carbenicillin added, and the culture was kept at 37 °C until reaching the desired optical density for induction. At induction point, IPTG was added to a final concentration of 500 μM, plus a second dose of carbenicillin, and the temperature was lowered to 15 °C. The culture was left for 18 h at 15 °C and then harvested. Cells were harvested by centrifugation and the cell paste resuspended in lysis buffer (50 mM Tris-Cl pH 8.0 100 mM NaCl) containing protease inhibitors (SigmaFast^TM^, Sigma, St. Louis, MO, USA). Cell rupture was performed by passing the cell paste twice through a cell disruptor (T5 1.1 kW, Constant Systems Ltd., Daventry, Northants, UK) at 25 kpsi. Cell free extract was clarified by centrifugation and addition of 1% Streptomycin Sulfate. Skylan-NS protein was purified from the supernatant using Ni-NTA affinity resin (Thermo Scientific, Waltham, MA, USA). The eluted protein was desalted using a Sephadex G25 column (GE Healthcare, Chicago, IL, USA) and eluted samples were kept at –20 °C before proceeding with further purification by gel filtration. Prior to gel filtration, samples were concentrated using Vivaspin 20 centricons (Sartorius, Göttingen, Germany) and then injected into a Superdex 75 column (GE Healthcare, Chicago, IL, USA) for separation in 50 mM Tris-Cl pH 8.0 100 mM NaCl 0.01% Sodium Azide buffer. Eluted samples were concentrated and buffer was exchanged to 10mM Tris-Cl pH 8.0 50 mM NaCl using Vivaspin 20 centricons.

Skylan-NS microcrystals were grown in 0.2 M lithium sulfate, 0.1 M Tris-Cl pH 8.5, 25–30% Poly-ethylene glycol (PEG) 3350, at final protein concentrations of 6 mg/mL or 9 mg/mL. For batch crystallization, concentrated stocks of protein and precipitant were mixed in 1:3 or 1:2 ratios in Eppendorf tubes, to achieve the final conditions listed above, using a similar free interface diffusion method to that described in Kupitz et al. [53]. The mix was incubated at 20 °C degrees for 24 h and needle shaped crystals were obtained with an average density of 1 × 10^9^ crystals/mL. Crystal density was calculated on diluted samples using a Neubauer counting chamber (Neubauer Improved Bright-line, Hirschmann, Eberstadt, Germany).

**Figure A1:**
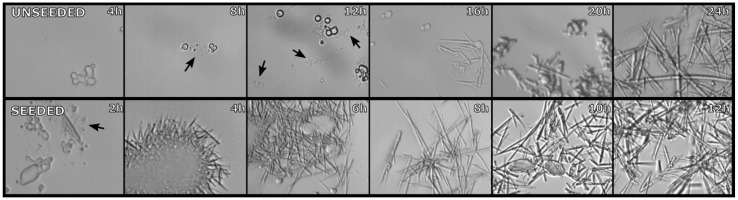
Images of samples taken from batch crystallization stocks at different growth times. In both un-seeded (upper panels) and seeded (lower) schemes. Arrows points to early crystal growth. All images taken with ×500 magnification.

Batch crystal growth appears to begin with the formation of small aggregates of precipitated protein from which initial nano/microcrystals form (arrows in Figure A1). These increase in size until aggregates are resolubilized and growth stops. Adjustment of the PEG and protein concentration can be used to control the final crystal size. While batch crystallization has a mostly uniform distribution of crystal size, some larger crystals are present, and these were gently separated from the slurry using gravity driven filters (CellTrics, Sysmex-Partec, Görlitz, Germany), with the aperture size depending on delivery mechanism. Seeding was shown to increase the speed of crystal formation but was less consistent (see Appendix A and Appendix B).

### 4.2. Crystal Injection

When using the grease injector, batch grown micro crystals were gently pelleted in a mini centrifuge and the supernatant removed. The concentrated slurry is them combined with a mineral oil–based grease matrix in an approximate 1:10 ratio. The grease crystal suspension is then loaded into a syringe and extruded in to the interaction region as a continuous stream. A suction nozzle was placed underneath the jet to collect debris and provide a degree of stretching and thinning of the grease column. The grease injector has the advantage of being very conservative of sample and a larger aperture allows crystals up to 150 µm in the longest dimension to be shot.

**Figure A2:**
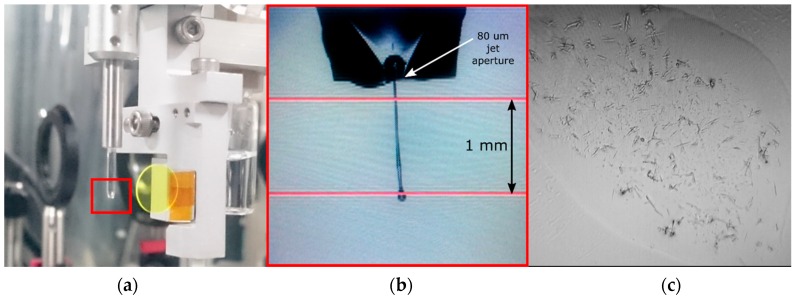
(**a**) Droplet injector assembly, (**b**) close up image of institute droplet back lit with strobed LED and (**c**) micro crystals contained in a single droplet collected on a microscope slide.

The droplet injector employs an acoustic inkjet head (IJHDS-1000, MICROJET (Shiojiri, Nagano, Japan)) driven by current pulses to deliver ≈20 μm thick droplets of crystal slurry on demand (Figure A2). The volume and size of the droplets can be controlled by varying the voltage and temporal width of the current pulse. During operation for SFX droplets are estimated to contain 100–200 microcrystals and the injector consumed ≈100 μL/h.

The GDVN consists of a needle placed inside a glass capillary through which helium flows. Crystal slurry is injected out of the central needle and the helium serves to focus the slurry into a thin jet. For the GDVN the crystal slurry was allowed to settle and the supernatant removed to increase the concentration to ≈1.5 × 10^9^ crystals/mL and filtered to a maximum crystal size of 10 µm.

During XFEL beam time the off-state was prepared by pre-illuminating the crystal slurry with several high power (750 mW each) 500 nm LED arrays for 10–20 min, pipetting up and down every 2 min. The sample was then kept in a less intense 500 nm light box until needed. A final 3 min of intense illumination was applied prior to loading. Sample was changed approximately every 3 h. As an added precaution and to ensure the 100% off-state when using the droplet injector a 60 mW 473 nm CW laser was focused onto the glass nozzle of the injector to back convert any thermal reversion that may have taken place. The LCLS data later showed that the CW laser was unnecessary as the crystals had not undergone any measureable thermal reversion, with a maximum of 3 h between samples loading.

### 4.3. Data Collection

Four distinct datasets were collected (Table 1) for this work. The X-ray parameters are listed in Table 2. Two different methods of focusing the XFEL beam were used; Kirpatrick-Baez mirror pairs [54,55] or Beryllium refractive lenses. SACLA beam time crystallographic data was collected using the “SWD-octal MPCCD” detector [33], a 4 MPx CCD based detector with eight segments arranged in a fixed geometry. The grease and droplet injector “on” state datasets were collected in Experimental Hutch 4 (EH4) using the DAPHNIS chamber [27] during a beam time where the photon energy and detector distance were fixed. The off-state droplet injector dataset was collected in EH2. SFX data was processed using the SACLA pipeline [51], built on Cheetah [52] and CrystFEL [30]. The off-state GDNV data was collected at LCLS; CXI using CSPAD detector. The data was processed using *Psana* [56] and *Psocake* [57] codes and CrystFEL [30]. All merging was performed using CrystFEL [30].

### 4.4. Structure Solution and Refinement

Structure factor amplitudes from the merged CrystFEL output files were obtained using Truncate [58]. Molecular replacement was conducted on all data sets using MOLREP [59] against Dronpa (PDB: 2GX2; [60]) and the model was iteratively refined using Refmac [61] with manual modeling and adjustments carried out in Coot [62]. Descriptions of the chromophore and links were generated with JLigand [63] and refined with Refmac. Electron density maps were calculated using FFT [64] and figures were prepared using PyMol [65]. Skylan-NS structures have been submitted to the Protein Database with codes, SACLA-on (grease) 5OOZ, SACLA-on (droplet) 5OQE, SACLA-off (droplet) 5OQ9 and LCLS-off (GDVN) 5OQA.

## 5. Conclusions

We have made a comparison of SFX data collected at stations at SACLA and at LCLS, for micro-crystals of a photochromic fluorescent protein ‘Skylan-NS’. We have additionally evaluated the data quality with using different injection methods Furthermore, the photoconversion of the ‘on-state’ to the ‘off-state’ with green illumination, which proceeds to completion, allowed the evaluation of isomorphous structure factor differences that were found to be larger than those arising from instrumentation and wavelength differences. We find that the data quality and limiting resolution is not affected by the injection method, comparing the droplet-on-demand, which involves high pressure shock waves for delivery, with the continuous flow ‘GDVN’ method used at LCLS. Photoconversion of ‘Skylan-NS’ does not degrade diffraction quality, and structural differences between the on- and off-states were found to resemble those occurring in Dronpa closely, with some differences with regard to amplitude of side-chain motion.

## Figures and Tables

**Table 1:** Crystallography statistics for the four Skylan-NS SFX data sets.

Dataset	SACLA-On (Grease)	SACLA-On (Droplet)	SACLA-Off (Droplet)	LCLS-Off (GDVN)
Wavelength (eV)	7036.69	7036.69	10500	9477.021
Resolution (Å)	39.8–1.911	54.31–1.911	39.80–1.45	54.31–1.533
Space group	P2_1_2_1_2_1_	P2_1_2_1_2_1_	P2_1_2_1_2_1_	P2_1_2_1_2_1_
Unit cell (Å)	39.8 74.6 79.2 90 90 90	39.8 74.6 79.2 90 90 90	39.8 74.6 79.2 90 90 90	39.8 74.6 79.2 90 90 90
No. of patterns	29,157	9,569	27,318	24,398
No. of merged patterns	18,582	6,902	22,008	11,275
Total reflections	3,353,434	1,195,672	4,483,117	2,616,480
Unique reflections	18,901	18,911	37,792	36,135
Completeness	99.74	96.8	90.4	78.3
Mean I/σ(I)	13.77	7.25	6.84	5.96
Wilson B-factor	29.934 (4.047 σ)	35.824 (9.820 σ)	28 (4.8 σ)	43.29
R-split	7.86	15.64	17.23	21.38
CC* correlation	0.9980	0.9895	0.9947	0.9835
Refls. used in refinement	18304 (1499)	17009 (525)	29593 (100)	27222 (70)
Refls. used for R-free	927 (78)	855 (23)	1471 (2)	1342 (3)
R-work (highest res)	0.1563 (0.7282)	0.1672 (0.4419)	0.1748 (0.6558)	0.1764 (0.6305)
R-free	0.2286 (0.7826)	0.2412 (0.5200)	0.2336 (0.5492)	0.2283 (1.2997)
No. of non-H atoms	1993	2017	1991	2060
macromolecules	1808	1819	1758	1809
ligands	23	23	23	23
solvent	162	175	210	228
protein residues	216	217	215	215
RMS (bonds)	0.018	0.016	0.021	0.025
RMS (angles)	1.93	1.89	2.15	2.09
Ramachandran favored (%)	98.10	98.11	99.53	99.05
Ramachandran allowed (%)	1.90	1.89	0.47	0.95
Ramachandran outliers (%)	0.00	0.00	0.00	0.00
Rotamer outliers (%)	3.98	4.43	3.16	3.98
Clashscore	4.97	6.83	7.12	8.50
Average B-factor	35.46	43.63	30.16	42.51
macromolecules	33.93	41.67	28.27	39.98
ligands	26.53	35.24	23.59	35.57
solvent	53.78	65.09	46.65	63.27

CC*; analytical estimate of true correlation coefficient (CC_true_) based on the Pearson correlation coefficient between random half-datasets (CC_1/2_).

**Table 2:** X-ray and detector parameters of collected datasets.

Dataset	SACLA-On (Grease)	SACLA-On (Droplet)	SACLA-Off (Droplet)	LCLS-Off (GDVN)
Wavelength (keV)	7.0	7.0	10.5	9.5
Pulse energy (mJ)	≈0.5	≈0.5	≈0.5	≈0.5
Focus method	KB *	KB *	Be ^†^	KB *
Spot size (μm)	1	1	1	1
Repetition rate (Hz)	30	30	30	120
Detector	MPCCD	MPCCD	MPCCD	CSPAD
Detector distance (mm)	50.000	50.000	66.941	99.998

* Kirpatrick-Baez mirror pair; ^†^ Beryllium lens stacks. MPCCD: Multi-Port Charge-Coupled Device; CSPAD: Cornell-SLAC Pixel Array Detector.

**Figure A3:**
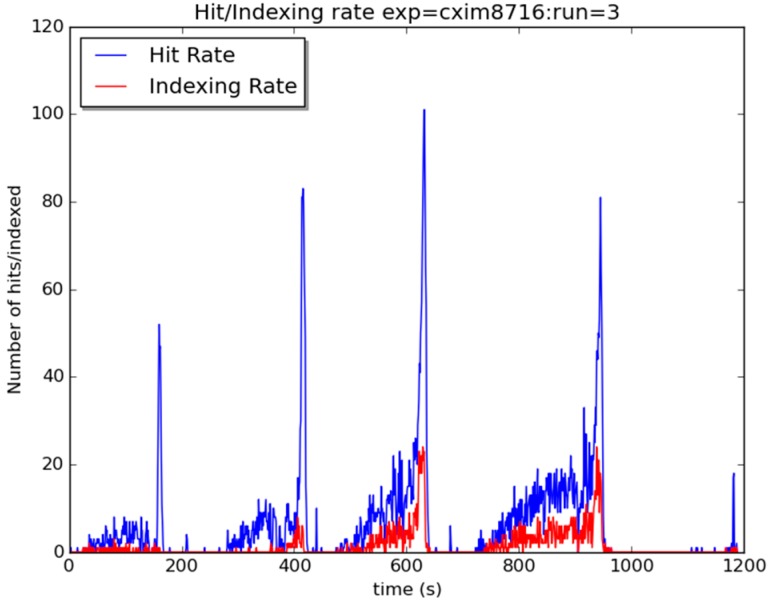
SFX hit (blue) and indexing (red) rate using GDVN at CXI, LCLS. Spikes in rates occurred after switch from sample to water.

## Abbreviations

SFX	Serial Femtosecond Crystallography
XFEL	X-ray Free Electron Laser
PCS	Protein Crystal Screening
LCLS	Linac Coherent Light Source
SACLA	SPring-8 Angstrom Compact free electron Laser

## Appendix A. Seeding Crystallization

Large crystals from plated methods were ground up and used as seeds for the batch crystallization. Approximately 40 μL of large crystals was combined with 50 μL of mother liquor and then ×10, ×100 and ×1000 dilutions were performed to make seed stocks. The ×10 and ×100 dilution seeds were found to greatly increase the speed of crystal formation with saturation being achieved within 12 h as compared to the 24 h in the unseeded (Figure A1). The ×1000 dilution showed little effect. For SFX it was decided to use unseeded growth due the inconsistency in preparing large quantities of seed stock. The standard crystallization conditions of final 6 mg/mL in 30% PEG 3350 gave a crystal concentration of 1 × 10^9^ crystal/mL.

## Appendix B. Crystal Delivery Complications

To collect images of the droplet injector, the nozzle was back-lit with a strobed LED, which allowed “freeze-frame” images of the droplet to be collected to assess the effect of mother liquor PEG concentration on droplet delivery. During XFEL beam time this setup was also used to monitor overlap between the droplets and XFEL beam. Due to the dry helium environment of the detector chamber, it was necessary to reduce the PEG concentration of the crystal slurry as much as possible (25%) to minimize drying and the collection of debris on the nozzle which impeded normal operation. This could not be fully eliminated and regular cleaning of the nozzle with distilled water was required whenever sample was changed.

GDVN sample delivery in viscous high-molecular-weight PEG was possible but required repeated switching between water and crystal slurry, using also the largest bore diameter available (200 μm). Pressure gradually built inside the injection lines when injecting the Skylan-NS slurry. Once it reached the maximum pressure of the system, it was necessary to switch to water to flush out the lines. This resulted in a very rapid increase in hit rate followed by a similarly rapid decrease (Figure A3). It is likely that this is due to the small diameter liquid lines used in this system and the relatively high PEG concentration (for this system) of the slurry which resulted in a large number of the crystals adhering to the inside of the lines and preventing flow. Once cleaning began these were all dislodged and released in one burst of highly concentrated slurry in the interaction region.
